# Feasibility of MRI attenuation correction in cardiac FDG-PET

**DOI:** 10.1186/1532-429X-15-S1-O61

**Published:** 2013-01-30

**Authors:** Jeffrey M Lau, Shivak Sharma, Richard Laforest, Jonathan McConathy, James Barnwell, Agus Priatna, Linda M Becker, Glenn J Foster, Robert J Gropler, Pamela K Woodard

**Affiliations:** 1 Cardiology, Washington University in St. Louis, Saint Louis, MO, USA; 2 Radiology, Washington University in St. Louis, Saint Louis, MO, USA; 3 Siemens, Malvern, PA, USA

## Background

Simultaneous acquisition PET-MRI is a new technology that has the potential to significantly impact diagnostic patient care. Cardiac imaging using PET-MRI offers high signal resolution MRI images superimposed on PET metabolic functional assessment. Specifically, 18F-fluorodeoxyglucose (FDG) PET-MR has the potential to provide both anatomic scar tissue evaluation and information regarding myocardial glucose metabolism. While early brain and soft tissue data have demonstrated that PET specific uptake values (SUVs) obtained using MRI for attenuation correction (AC) are comparable to SUVs obtained using CT AC, SUV measurements of myocardial tissue have not been compared. The objective of this pilot study is to determine the reproducibility of SUVs obtained by PET imaging using an AC µ-map comprised of a dual echo VIBE Dixon MRI sequence instead of CT.

## Methods

30 patients with no known cardiac history underwent full body PET-CT imaging (Siemens Biograph 40), followed by full-body simultaneous PET-MRI imaging (Siemens Biograph mMR). A single dose (10-15 mCi) of 18F-FDG radiotracer was injected on average 59 +/- 7 minutes prior to PET-CT image acquisition, and127 +/- 23 minutes prior to PET-MRI image acquisition. Images were whole body images acquired without cardiac gating. For PET-MR the AC µ-map was a dual echo VIBE Dixon sequence that separates water and fat (TE1/TE2 = 1.23 msec/2.46 msec, TR = 3.6 msec, left-right FOV = 500 mm, anterior-posterior FOV = 300 mm). Average SUVs were obtained by tracing the entire cross section of the left ventricular myocardium in the short axis of the heart at the papillary muscle level, using the syngo TRUE-D computer software (Siemens).

## Results

There is no statistically significant difference between the average SUVs obtained by PET-CT and PET-MRI (4.62 vs. 4.68, p=0.47). Although FDG uptake rate and the SUVs are highly variable among this study group, there is excellent per patient correlation between the values acquired by PET-CT and PET-MRI (R2 =0.97, slope = 0.78, Figure).

**Figure 1:**
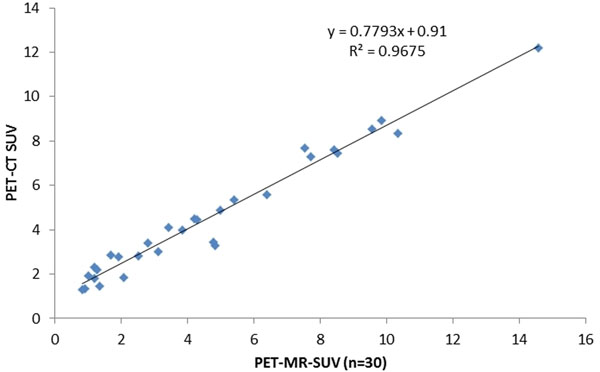
PET-MRI and PET-CT SUVcorrelation in normal myocardium

## Conclusions

Myocardial PET SUVs measured using MRI as AC show excellent correlation to those obtained by standard PET-CT imaging. Future studies will focus on the optimization of cardiac-gated simultaneous PET-MRI image acquisition.

## Funding

Mallinckrodt Institute of Radiology Departmental Funds.

